# Effect of Light Intensity and Wavelength on Biomass Growth and Protein and Amino Acid Composition of *Dunaliella salina*

**DOI:** 10.3390/foods10051018

**Published:** 2021-05-07

**Authors:** Yixing Sui, Patricia J. Harvey

**Affiliations:** Aquatic Biotechnology and Biology, Faculty of Engineering and Science, University of Greenwich, Central Avenue, Chatham Maritime, Kent ME4 4TB, UK; y.sui@gre.ac.uk

**Keywords:** microalgae, protein source, photobioreactor, microbial protein, light quality

## Abstract

*Dunaliella salina* is a halotolerant, photoautotrophic marine microalga and one of the richest sources of natural carotenoids but also shows potential as a novel food source with high protein quality. This study sought to optimise the production of biomass, protein and amino acids from *D. salina*, alongside carotenoids using a two-stage cultivation approach based on the use of light of different intensities and quality, i.e., white, red and blue LED light. In stage 1, four white LED light intensities were tested. In stage 2, the same four light intensities from either blue or red LEDs were applied once exponential growth ceased and cells reached the stationary phase under white LED light in stage 1. Remarkably, both biomass concentration and biomass productivity showed a 1.3–1.7-fold increase in stage 2, without medium replenishment, while protein concentration and protein productivity showed an ~1.1-fold increase. The amino acid content and amino acid index remained unchanged from stage 1 to stage 2, and minimum difference was found across different light intensities. Overall, *D. salina* delivered so-called high protein quality, with an essential amino acid index (EAAI) of 0.99, and red light, which has previously been shown to increase carotenoid production, boosted further biomass production over and above white light, at all light intensities tested.

## 1. Introduction

Microalgae have been proposed as a sustainable protein source for human food since the early 1950s, owing to their high protein content and well-balanced protein quality. They supply essential amino acids (EAA), which cannot be synthesised by humans [1,2]. Many microalgal species and strains have revealed a comparable, or even better, EAA profile than conventional protein sources (e.g., soybean and egg), which moreover fulfils human requirements referenced by the FAO/WHO [2,3]. Historically, *Dunaliella salina* has been studied and exploited mainly for its high β-carotene content. This alga is one of the richest sources of β-carotene, which is currently sought after on worldwide markets as a food colorant [4]. In *D. salina*, β-carotene accumulates to levels of up to 10% of the dry biomass, depending on the integrated amount of light, especially red light, to which the alga is exposed during a cell division cycle [5]. β-carotene, especially *9-cis* β-carotene [5], is also amongst the most potent of carotenoids needed in the human diet to produce retinoids; these play important roles in cell differentiation, growth and apoptosis and are critical for controlling vision defects [6,7]. However, the potential of *D. salina* to be used as a protein source has gained increased attention in recent years, even after separation of carotenoids [6,8]. Depending on cultivation conditions, *D. salina* possesses a protein content of 57% (dry weight (DW) basis) or up to 80% on an ash-free dry weight (AFDW) basis [2,9]. The overall protein quality of *D. salina*, as shown in EAA content and essential amino acid index (EAAI), also illustrates its suitability as a high-quality protein source [2,3,10,11]. Nevertheless, several factors such as nutrient limitation, growth stage and light intensity significantly impact EAA synthesis in *D. salina*. For instance, nitrogen limitation resulted in enhanced production of all EAAs in *D. salina* CCAP 19/18 [11]; sulphur deprivation resulted in increased valine, leucine and threonine content in *D. salina* TG [12]; and phosphorus deprivation also enhanced the content of most EAAs in *D. salina* TG [13]. Apart from nutrient levels, higher light intensity seems to favour the production of most EAAs except for threonine in *D. salina* CCAP 19/18, yet delivers a different EAA profile compared with nitrogen limitation [11].

Light-emitting diodes (LEDs) of different wavelengths offer opportunities for delivering efficient, durable, reliable, economic and controllable light in the cultivation of microalgae, and their use has resulted in increased production of biomass and biomolecules [14,15]. In *D. salina*, different light wavelengths have been studied regarding their effects on carotenoids’ synthesis [5,16,17,18,19,20]. Red light enhanced the production of total carotenoids compared to blue, white and mixed red-blue light [20]; a higher content of *9-cis* β-carotene, which holds potential in the treatment of retinal dystrophies and other diseases, was found under red light [19]; red light increased the production of colourless carotenoids under conditions favouring their accumulation [5]; and, by using a blue-red light-shifting strategy, enhanced β-carotene production was achieved in *D. salina* compared to either blue, red or white light alone [17]. Nevertheless, the effect of different LED light wavelengths and intensities on protein and amino acid synthesis in *D. salina* has not yet been investigated. To fill this gap, this study applied red, blue and white light using a two-stage cultivation strategy to evaluate biomass, protein and EAA levels in *D. salina*.

## 2. Materials and Methods

### 2.1. Microalgal Strain and Cultivation Methods

**Figure 1:**
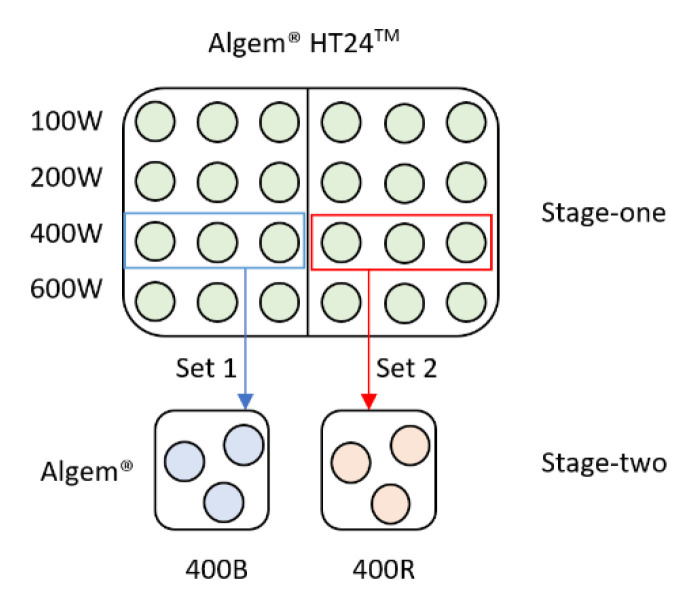
Scheme to illustrate the reactor setup of the two-stage experimental design. W = white LED light; B = blue LED light; R = red LED light. The associated numerical values refer to applied light intensity (µmol photos m^−2^ s^−1^). All stage 1 treatments were transferred to stage 2. The framed treatments from 400W to 400B and 400R are for demonstration purposes only.

*D. salina* CCAP 19/41 (PLY DF15) was obtained from the Marine Biological Association (MBA, Plymouth, UK). Sterilised modified Johnson’s medium at pH 7.5 [21] was used for cultivation. A two-stage cultivation approach was applied in the experiment (Figure 1 and Table 1). Two sets of four treatments with different white LED light intensities were used in stage 1. When the cultures reached the stationary phase around day 26, the two sets of treatments were transferred to blue- and red-light treatments separately in stage 2. For the two stage 2 treatments, blue or red LED light of the same light intensity as stage 1 treatments, was applied for 24 h. All treatments were performed in triplicates in 50 mL Erlenmeyer flasks aiming at inoculating concentration of 0.02 optical density at 740 nm (OD740). Algem^®^ HT24^TM^ and Algem^®^ photobioreactors from Algenuity (https://www.algenuity.com/, accessed on 15 March 2021) were used in stage 1 and stage 2 cultivations, respectively. The temperature was controlled at 25 °C, and the mixing was provided by 200 rpm orbital shaking.

### 2.2. Biomass Analyses and Calculations

Cell growth in stage 1 was monitored with built-in optical density data capture at 740 nm (OD740) from Algem^®^ HT24^TM^. At the end of both stages, samples were taken for biomass analysis. Biomass ash-free dry weights (AFDWs) were determined by filtrating 0.8 to 2.5 mL of algal suspension (depending on the cell density in each treatment) through pre-cleaned glass fibre filters (Whatman GF/F, Ø 25 mm, pore size 0.7 µm). Filters containing the samples were dried at 105 °C overnight in an oven (Fistreem International Ltd., Cambridge, UK), and the weights were recorded after cooling down in a desiccator. The filters were subsequently transferred to a 550 °C oven (Vecstar Ltd., Chesterfield, UK) for 2 h, after which the weight differences were determined gravimetrically. Protein content was determined using a Thermo Scientific™ Pierce™ modified Lowry protein assay kit. For full recovery of protein content from *D. salina* biomass, a 1 h incubation using 0.2 M NaOH at 40 °C was performed before protein analysis. Prior to amino acid analysis, pelletised biomass (5 min at 5000 g) was hydrolysed with 6 M HCl in a vacuum-sealed ampule glass tube for 24 h at 110 °C. The hydrolysed samples were directly prepared for GC-FID (Agilent 6850, Stockport, UK) separation and detection using the Phenomenex EZ: faast amino acid analysis kit [22]. Bovine serum albumin (BSA) was used as a control to calculate the amino acid recovery. Amino acid analysis was performed on one of the triplicates in each treatment. The essential amino acid index (EAAI) was calculated using FAO/WHO-established adult indispensable amino acid requirements as a reference following this equation:

(1) $$EAAI=\sqrt[{n}]{\frac{aa1}{AA1}\times \frac{aa2}{AA2}\times \ldots \ldots \times \frac{aan}{AAn}}$$

where *aan* and *AAn* are the EAA content (mg EAA g^−1^ protein) in the sample and FAO/WHO reference, respectively. The protein quality of the sample was categorised as high (EAAI > 0.95), good (0.86 < EAAI ≤ 0.95), useful (0.75 < EAAI ≤ 0.86) and inadequate (EAAI ≤ 0.75) [3]. The biomass, protein and EAA productivities (mg L^−1^ day^−1^) were derived from their concentration (g L^−1^) divided by the time of cultivation (days). Increase factors of all parameters were calculated as:

(2) $$Increase\;factor=\frac{P_{2}}{P_{1}}$$

where *P*_2_ is the parameter value obtained from blue- or red-light treatment in stage 2 (after 24 h LED exposure) and *P*_1_ is the parameter value obtained at the end of white-light treatments (26 days of LED exposure) in stage 1.

### 2.3. Statistics

The experiment was conducted in biological triplicates, with results expressed as the mean ± standard deviation in tables and figures (except for EAA results). The values stated in the main text are without the standard deviation for better readability. An independent sample *t*-test (to compare two groups) and one-way ANOVA followed by post hoc Tukey’s test (to compare multiple groups) were performed using IBM SPSS Statistics 26. Statistical difference was considered when *p* < 0.05.

## 3. Results

### 3.1. Biomass Growth

**Figure 2:**
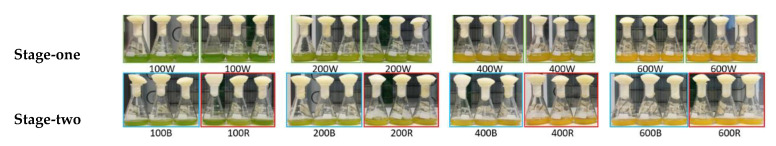
Appearance of *D. salina* cultures at the end of each cultivation stage. Cultivation was conducted at 25 °C, pH 7.5 and 200 rpm mixing, and cultures were maintained under white (W), blue (B) or red (R) LED light at light intensities of 100, 200, 400 or 600 µmol photos m^−2^ s^−1^.

**Figure 3:**
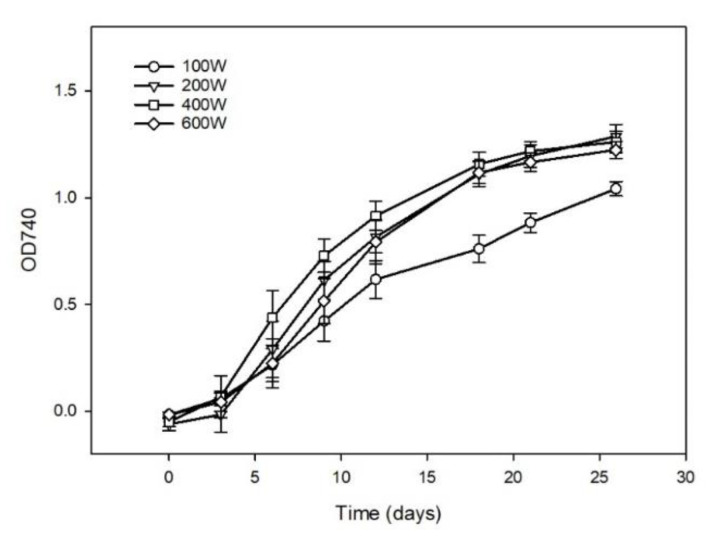
Growth curve at OD740 of *D. salina* during stage 1 under different light intensities using white LED light. Cultivation was conducted at 25 °C, pH 7.5 and 200 rpm mixing. Data are expressed as means ± standard deviation (*n* = 6).

Culture appearance and growth curves representing *D. salina* biomass accumulation in stage 1 are shown in Figure 2 and Figure 3. During the 26 days of cultivation in stage 1, microalgal cells profited initially from higher white-light intensities, resulting in higher biomass concentrations of 1.49, 1.30 and 1.43 g AFDW L^−1^ in 200W, 400W and 600W by day 26, respectively, compared with 0.89 g AFDW L^−1^ in 100W (Figure 3). Light availability limited the growth rate of the cells because initial growth rates increased with increasing light intensity, but slowed as cell densities increased and caused shadowing effects so that the number of photons received per cell was reduced (Figure 3).

Remarkably, switching from high-energy white LED light at the end of stage 1 cultivation (cell growth rate limited by light availability due to shadowing) to either blue or red LED light in stage 2 enhanced cell growth and resulted in a much higher biomass concentration and biomass productivity (Table 2). On average, in red LED light, biomass concentrations across four red-light treatments increased 1.69-fold within 24 h. The greatest increase in biomass concentration occurred under 400R red-light intensity (1.95-fold) and reached 2.50 g AFDW L^−1^ (Table 2). Interestingly, the greatest increase in biomass concentration for blue-light treatments was also under 400 µmol photons m^−2^ s^−1^ blue-light intensity, and the biomass concentration reached 2.03 g AFDW L^−1^ (1.59-fold the value measured at the end of stage 1 cultivation), with an average increase of 1.37-fold across four treatments compared to stage 1. The increase in biomass productivities in stage 2 reflected similar effects of light limitation, i.e., increased rates of growth with increasing light intensity, until effects of cell shadowing reduced the light availability and the biomass productivity fold-increase that could be achieved (Table 2). In stage 2, red LED light delivered significantly higher levels of both biomass concentration and biomass productivity compared to blue LED light (*p* < 0.05).

### 3.2. Protein Content and Protein Concentration Dynamics

**Figure 4:**
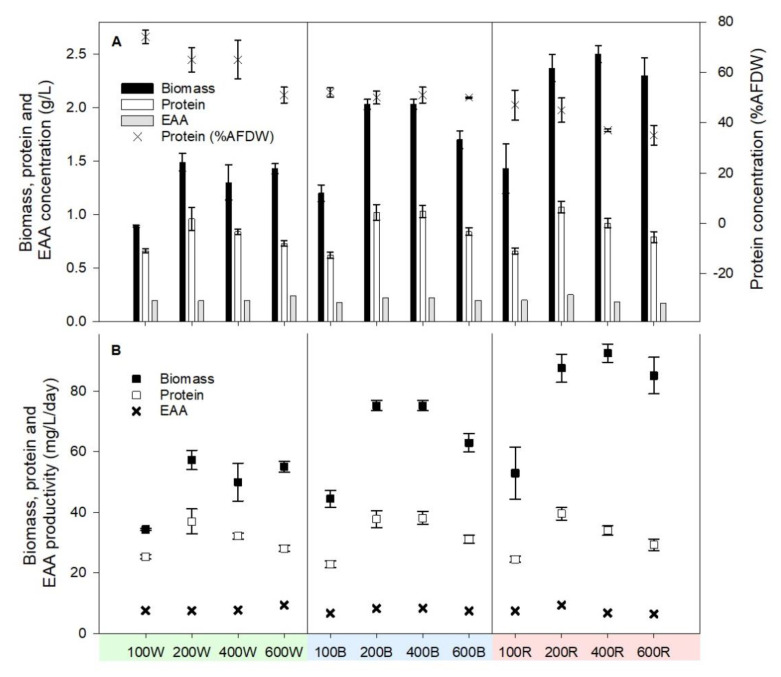
Biomass, protein and, EAA concentration (**A**) and biomass, protein and EAA productivity (**B**) of *D. salina* at the end of both stage 1 (26 days) and stage 2 (+24 h) under different light intensities and wavelengths. Cultivation was conducted at 25 °C, pH 7.5 and 200 rpm mixing. Data are expressed as means ± standard deviation (*n* = 3) except EAA data.

In stage 1, protein content relative to the ash-free dry weight decreased with increasing light intensity from 74% AFDW under 100W to 52% AFDW under 600W after 26 days (Figure 4). In stage 2, protein content under each of the four different blue-light intensities remained at around 50% AFDW, while under red light, the protein content (%AFDW) decreased further with increasing light intensity: protein represented 47% AFDW under 100R and 35% AFDW under 600R (Figure 4). Compared with stage 1, blue- and red-light treatments in stage 2 reduced the protein content, relative to the ash-free dry weight, 0.81- and 0.65-fold with the greatest reduction in 100B (from 74% AFDW to 52% AFDW) and 400R (from 65% AFDW to 37% AFDW), respectively (Table 2). Because of the increased biomass concentration (g AFDW L^−1^) and decreased protein content (%AFDW) in stage 2 compared to stage 1, the protein concentration (g L^−1^) and protein productivity (mg L^−1^ day^−1^) mostly remained the same across all treatments in stage 2. There was no significant difference (*p* < 0.05) between stage 1 white- and stage 2 blue- and red-light treatments (Figure 4); hence, the increase factors for blue- and red-light treatments were all close to 1 (Table 2). The highest protein productivity among all treatments was achieved under 200R, at 40 mg L^−1^ day^−1^; this treatment also delivered the highest protein concentration of 1.07 g L^−1^ and a protein content of 45% AFDW (Figure 4).

### 3.3. Amino Acids

**Figure 5:**
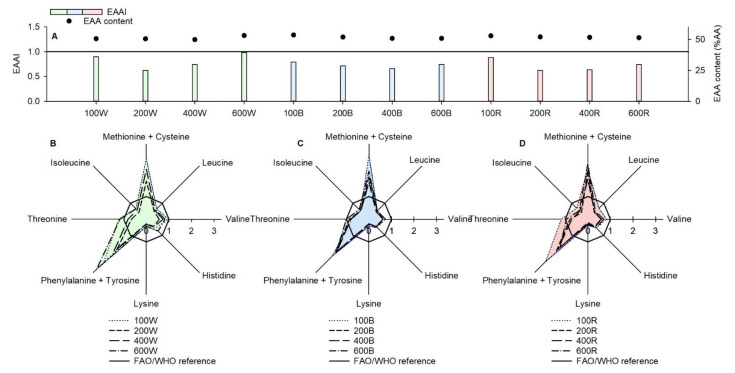
EAA content, EAAI (**A**) and individual EAA level of *D. salina* at the end of both stage 1 (**B**) and stage 2 (**C**,**D**) under different light intensities and wavelengths. Cultivation was conducted at 25 °C, pH 7.5 and 200 rpm mixing.

The overall protein quality of *D. salina*, as indicated by the essential amino acid index (EAAI), from both stage 1 and stage 2 treatments varied from inadequate to high, with high protein quality achieved under 600W (EAAI = 0.99), good under 100W (EAAI = 0.90) and 100R (EAAI = 0.89), useful under 100B (EAAI = 0.79) and inadequate under the rest (EAAI ≤ 0.75) (Figure 5). The EAA content represented roughly 50% over the total AA content across all treatments (Figure 5). The individual EAA distribution across all treatments was broadly similar (Figure 5). High levels of methionine + cysteine and phenylalanine + tyrosine were produced in *D. salina*, yet the rest of EAAs were limiting with reference to FAO/WHO requirements (Figure 5). The high quality protein derived from 600W had a total EAA content of 340.1 mg g^−1^ protein, which was 1.14-fold higher than the 271 mg g^−1^ protein required by the FAO/WHO (Figure 5 and Table 3). In addition, 100W, 100B and 100R delivered a higher total EAA content than FAO/WHO requirements (Table 3). Nevertheless, some individual EAA contents of *D. salina* in these treatments were greatly limiting. Histidine, isoleucine and leucine contents were around half of the required level, and the lysine content was even around one-fourth of the required level, which compromised the individual EAA quality in *D. salina* (Table 3). When looking at variations of the individual EAA profile and content within each treatment in Table 3, it is difficult to draw solid conclusions, as statistics showed no significant difference among them (*p* > 0.05). For instance, higher phenylalanine + tyrosine at 100R (100.3 mg g^−1^ protein) was observed when compared with other red-light treatments (65.8–74.7 mg g^−1^ protein), yet was statistically insignificant (Table 3). For both EAA concentration and EAA productivity, there was also no significant difference (*p* > 0.05) among all treatments from stage 1 and stage 2, showing increase factors close to 1 (Table 2).

## 4. Discussion

### 4.1. Effect of Light Intensity and Wavelength

Different microalgae show different responses in biochemical composition towards light intensity. On the one hand, cellular protein in *D. salina* CCAP 19/30 remained at similar levels among various light intensities ranging from 50 to 1500 µmol photos m^−2^ s^−1^ [23]. Similar protein contents were also found with *Chlorella vulgaris* across light intensities from 130 to 520 µmol photos m^−2^ s^−1^, despite changes in lipid contents [24]. On the other hand, with increasing light intensity, some *Dunaliella*, *Chlorella* and *Scenedesmus* species showed reductions in protein content [25,26,27], while others showed increased protein and carotenoids content [11]. High light intensity generally causes stress in microalgae, inhibiting photosynthesis, and directs carbon and energy flows towards the synthesis of reduced forms of storage compounds rather than protein [25,26]. Nonetheless, the stress impact depends on the microalgal species and specific light intensity. In *D. salina* CCAP 19/30, a decreased photosynthetic rate was observed for cultures acclimated to light intensities of 200 and 500 μmol photons m^−2^ s^−1^ due to photoinhibition, but at higher light intensities, intracellular glycerol increased and stabilised the photosynthetic apparatus, such that the photosynthetic rate increased to a maximum level [23].

Red light and blue light have been proven to have various effects on the biomass growth and biochemical composition of *D. salina* and other microalgae. Red light was the most efficient for *D. salina* growth compared to white and blue light and also resulted in the highest level of β-carotene accumulation at 25.21 μM [17,20]. Using the same *D. salina* DF15 strain, previous studies from our laboratory found no difference in cell density during 7-day cultivation under white, blue, red, red/blue 1/1, red/blue 1/2 and red/blue 2/1 at 1000 µmol photos m^−2^ s^−1^, yet red light led to the highest level of total carotenoids, β-carotene, *9-cis* β-carotene, carotenoids/chlorophyll ratio, *9-cis* β-carotene/β-carotene ratio and *9-cis*/*all-trans* β-carotene ratio [19,20]. Higher red-light intensity also resulted in higher levels of total carotenoids, *9-cis* β-carotene, carotenoids/chlorophyll ratio and *9-cis*/*all-trans* β-carotene ratio across 200, 500 and 1000 µmol photos m^−2^ s^−1^ [19,20], and these effects can be visually distinguished in the present study by the brighter-orange colour at higher/red light in comparison to lower/white and blue light (Figure 2). One-stage cultivation compared to a two-stage approach based on adoption of different light wavelengths, as described here, differs in its effects on microalgal growth and its composition. Han et al. (2019) [17] tested the effect of white, red and blue wavelengths on the cell growth of *D. salina* in one-stage growth, as well as a two-stage shift from blue to red light using normal and blue-light adapted cells. Their two-stage shift approach (6 days blue light and 5 days red light) enhanced both biomass and the β-carotene concentration compared with all one-stage approaches, and blue-light-adapted cells had the highest production of biomass and β-carotene during the 11-day cultivation period [17]. A LED shift strategy from red to blue wavelength greatly enhanced the astaxanthin production in *Haematococcus pluvialis* compared with either continuous red or blue light [28]. Sui et al. (2019) [11] tested light intensity and nitrogen content in a two-stage approach, where *D. salina* CCAP 19/18 had enhanced production of protein and carotenoids simultaneously. When nitrogen is sufficient in stage 1, higher light intensity in stage 2 without medium replenishment also promotes the cell volume increase in *D. salina*, which potentially contributes to an increase in biomass concentration [11]. This is in line with the findings in this study, which highlighted the effect of the light spectrum rather than nutrient replenishment. The emission spectrum of the red LED light used in the present work (625–680 nm) emits photons with the exact range required by molecules of chlorophyll-a and chlorophyll-b for photosynthesis, whereas mainly chlorophyll-b absorbs blue light (430–465 nm) [7,18,29]. *D. salina* has a higher chlorophyll-a content than chlorophyll-b, which is the primary electron donor for photosynthesis [30]. However, the production of carotenoids under red light is coupled with oxygen reduction and consequently alleviates the potential for photoinhibition under red light. By contrast, under blue light, carotenoid over-production coupled with oxygen reduction is precluded and photoinhibition ensues [5,19,20]. Higher biomass production under red light compared to under blue light is therefore to be expected, as demonstrated in this study (Figure 4 and Table 2).

### 4.2. Amino Acid Dynamics

The amino acid content of microalgae varies significantly across species and strains, as shown with 38 screened microalgae [31]. Particularly, limited information is available regarding the amino acid contents in *D. salina* and the variation in amino acids as affected by cultivation conditions [8]. Becker (2007) [2] and Kent et al. (2015) [3] demonstrated generally good protein quality of several microalgal species, including *Dunaliella bardawil* and a *Dunaliella* sp. For *D. salina* SAG 184.80, a later growth stage associated with nitrogen limitation under 24 h continuous light resulted in an increase in all EAA contents, compared to a 12/12 h light/dark regime [10]. A similar result was also observed for *D. salina* CCAP 19/18, where both EAA and carotenoid contents were simultaneously enhanced using a two-phase cultivation strategy based on a combination of nitrogen limitation and light intensity: in phase 1, nitrogen limitation increased the EAA content, and phase 2, which comprised higher illumination under nitrogen limitation, boosted carotenoid production [11]. Microalgal cells typically respond to nitrogen limitation by redirecting their metabolism to increase the degradation of certain amino acids and repress the biosynthesis pathways of others; glutamate levels may decline along with the cellular amino acid pool, but stress-related amino acids such as proline, lysine and tyrosine may increase [10,32,33,34,35]. Carbon and energy flows are typically directed towards TAG accumulation [32]. Under these conditions, the EAAI will improve. Phosphorus and sulphur limitation also alters metabolism in *D. salina*, resulting in increased contents of storage and stress-related compounds, including proline, lysine, polyamines such as cadaverine and antioxidants such as L-ascorbic acid and L-methionine, which will improve the EAAI [12,13,36].

The effect of light wavelength has been studied on limited microalgal species regarding their amino acid synthesis and has not been tested on *D. salina*. The concentration of essential and non-essential free amino acids in *Spirulina* sp. LEB 18 was higher when cultivated under green and blue light, while red light positively contributed to the formation of non-essential amino acids [15]. When a shifting light strategy from red light to blue light was provided to *Phaeodactylum tricornutum*, the pool of amino acid content was elevated within 30 min and then decreased after 24 h; when the strategy was from blue light to red light, all amino acid contents quickly decreased after only 15 min [37]. Jungandreas et al. (2014) [37] related the synthesis of amino acids to the enzymatic activity of pyruvate kinase (PK), which catalyses the formation of pyruvate as a central metabolite of the tricarboxylic acid cycle (TCA) cycle. After switching from red light to blue light, the turnover rate of PK in *P. tricornutum* increased, while switching from blue light to red light decreased PK activity. Conversely, in *Nannochloropsis gaditana*, red filtered light strongly enhanced the concentration of amino acids, especially asparagine (13-fold increase) [38]. Pyruvate carboxylase activity also increased, consistent with active amino acid biosynthesis under red light and the need to replenish TCA intermediates during this process. Under blue light, only the stress-related amino acid proline increased. Although detailed metabolites were not determined in the current study, our preliminary results indicated that no significant changes in the content of EAA were found in *D. salina* after 24 h under either blue or red light alone. However, the effect of light regimes, including light intensity, light wavelengths and light period, on the biosynthesis of amino acids and other nitrogen metabolites requires further investigation. Interestingly, when higher-value carotenoids were separated from *D. salina* biomass prepared under industrial conditions, the residue also presented a good essential amino acid and lipid profile to be used for food [6]. This ties in with Sui et al. (2019) [11] on the potential of co-producing high-value carotenoids and high-quality protein simultaneously in *D. salina* in a sustainable and efficient manner.

## 5. Conclusions

The two-stage cultivation approach using different light intensities and wavelengths enhanced both biomass concentration and biomass productivity of *D. salina*, where red light, which also increased the carotenoid content, was found to be more beneficial than blue light. In stage 2, the protein concentration and protein productivity increased slightly under both red and blue light after 24 h, whilst the EAA profile, EAA content and EAAI mostly remained unchanged without medium replenishment. *D. salina* delivered high protein quality under 600W, good protein quality under 100W and 100R and useful protein quality under 100B. Further studies will aim to combine the nutrient supply regime and the light regime together to optimise the biomass growth and protein and amino acid composition in *D. salina*.

## Figures and Tables

**Table 1:** Light regimes of the two-stage experimental design.

	Stage 1 White (W) Light				Stage 2 Blue (B) and Red (R) Light			
Treatments	100W	200W	400W	600W	100B	200B	400B	600B
					100R	200R	400R	600R
Light intensity (µmol photos m^−2^ s^−1^)	100	200	400	600	100	200	400	600

**Table 2:** Increase factors of blue- and red-light treatments in stage 2 (after 24 h LED exposure) compared to values obtained at the end of white-light treatments (26 days of LED exposure) in stage 1.

	100B	200B	400B	600B	100R	200R	400R	600R
AFDW concentration	1.34 ± 0.07	1.37 ± 0.05	1.59 ± 0.20	1.19 ± 0.07	1.60 ± 0.25	1.59 ± 0.08	1.95 ± 0.23	1.61 ± 0.16
Avg *^,a^	1.37 ± 0.18				1.69 ± 0.25			
Protein concentration	0.94 ± 0.05	1.07 ± 0.09	1.23 ± 0.05	1.16 ± 0.03	1.00 ± 0.06	1.12 ± 0.12	1.10 ± 0.02	1.08 ± 0.08
Avg *	1.10 ± 0.12				1.08 ± 0.09			
%AFDW Protein	0.70 ± 0.00	0.78 ± 0.07	0.79 ± 0.13	0.98 ± 0.06	0.64 ± 0.07	0.70 ± 0.06	0.57 ± 0.07	0.69 ± 0.12
Avg *^,b^	0.81 ± 0.13				0.65 ± 0.10			
EAA concentration	0.91	1.14	1.12	0.81	1.13	1.14	0.82	0.87
Avg *	1.00 ± 0.14				0.99 ± 0.21			
Biomass productivity	1.29 ± 0.07	1.32 ± 0.05	1.53 ± 0.19	1.14 ± 0.07	1.54 ± 0.24	1.53 ± 0.08	1.88 ± 0.22	1.55 ± 0.15
Avg *^,c^	1.32 ± 0.18				1.62 ± 0.24			
Protein productivity	0.90 ± 0.05	1.03 ± 0.09	1.19 ± 0.05	1.11 ± 0.03	0.97 ± 0.06	1.08 ± 0.12	1.06 ± 0.02	1.04 ± 0.07
Avg *	1.06 ± 0.12				1.04 ± 0.09			
EAA productivity	0.88	1.10	1.08	0.78	0.99	1.25	0.89	0.69
Avg *	0.96 ± 0.13				0.95 ± 0.20			

* Averaged from all four blue- or red-light treatments. ^a^ *p* = 0.002; ^b^ *p* = 0.003; ^c^ *p* = 0.002.

**Table 3:** Individual and total amino acid content of selected treatments and the FAO/WHO reference.

mg/g Protein	Histidine	Isoleucine	Leucine	Lysine	Methionine + Cysteine	Phenylalanine + Tyrosine	Threonine	Valine	Total EAA
100W	8.3	19.7	37.9	9.7	58.6	101.4	27.9	37.9	309.3
200W	6.7	13.7	28.8	9.2	37.1	68.8	15.3	22.1	206.6
400W	7.1	17.2	32.3	10.4	48.5	78.1	18.7	28.7	247.2
600W	11.1	19.5	34.8	14.1	73.7	118.4	26.9	32.0	340.1
100B	7.8	15.7	31.0	13.7	62.3	87.8	17.6	28.1	271.1
200B	7.0	16.0	30.8	10.6	41.6	80.6	19.9	24.5	236.3
400B	6.7	15.4	27.2	9.0	36.0	75.9	18.6	24.5	218.4
600B	6.8	18.3	31.0	9.2	46.6	77.6	23.0	28.8	247.3
100R	8.1	20.6	40.6	12.2	48.3	100.3	26.9	32.3	295.3
200R	6.9	12.0	23.9	6.6	47.3	74.7	18.1	24.1	219.4
400R	6.5	15.0	25.2	9.7	39.1	71.2	16.3	23.0	211.3
600R	8.0	16.0	28.1	10.8	53.9	65.8	21.8	27.6	239.5
FAO/WHO	15	30	59	45	22	38	23	39	271

Underlined values indicate levels above the FAO/WHO reference.
